# Prussian blue technique is prone to yield false negative results in magnetoreception research

**DOI:** 10.1038/s41598-022-12398-9

**Published:** 2022-05-25

**Authors:** Franziska Curdt, Katrin Haase, Laura Ziegenbalg, Helena Greb, Dominik Heyers, Michael Winklhofer

**Affiliations:** 1 AG Sensory Biology of Animals, Institute of Biology and Environmental Sciences, University Oldenburg, 26111 Oldenburg, Germany; 2 AG Neurosensorics, Institute of Biology and Environmental Sciences, University Oldenburg, 26111 Oldenburg, Germany; 3 Research Centre for Neurosensory Sciences, University of Oldenburg, 26111 Oldenburg, Germany

**Keywords:** Somatosensory system, Histology, Risk factors

## Abstract

Perls’s Prussian blue staining technique has been used in magnetoreception research to screen tissues for iron-rich structures as proxies for putative magnetoreceptor structures based on magnetic particles. However, seemingly promising structural candidates in the upper beak of birds detected with Prussian blue turned out to be either irreproducible or located in non-neuronal cells, which has spurred a controversy that has not been settled yet. Here we identify possible pitfalls in the previous works and apply the Prussian blue technique to tissues implicated in magnetic-particle-based magnetoreception, in an effort to reassess its suitability for staining single-domain magnetite, i.e., the proposed magnetic substrate for the interaction with the external magnetic field. In the upper beak of night-migratory songbirds, we found staining products in great numbers, but not remotely associated with fiber terminals of the traced ophthalmic branch of the trigeminal nerve. Surprisingly, staining products were absent from the lamina propria in the olfactory rosette of rainbow trout where candidate magnetoreceptor structures were identified with different techniques earlier. Critically, magnetosome chains in whole cells of magnetotactic bacteria remained unstained. The failure to label single-domain magnetite in positive control samples is a serious limitation of the technique and suggests that two most influential but antipodal studies conducted previously stood little chances of obtaining correct positive results under the assumption that magnetosome-like particles were present in the tissues. Nonetheless, the staining technique appears suitable to identify tissue contamination with iron-rich fine dust trapped in epithelia already in vivo.

## Introduction

Numerous behavioral experiments have demonstrated the ability of migratory animals to orient by the Earth’s magnetic field, but the nature of the underlying magnetic sensory structures remains one of the greatest mysteries in sensory biology^1^. The magnetic sense has been studied most thoroughly in night-migratory songbirds, where various lines of evidence point towards the existence of at least two fundamentally different magnetoreception mechanisms. One is based on radical pairs, with the flavoprotein cryptochrome 4 as the potential magnetic sensory molecule, located in cone photoreceptor cells in the retina^2–5^ and suggested to provide directional, i.e., “compass” information^6^. The radical-pair mechanism is consistent with the observation that songbirds tested in Emlen funnels were magnetically disoriented during exposure to weak radiofrequency magnetic fields aimed at interfering with the mechanism^7–9^ (but see Bojarinova et al. 2020^10^). The second magnetoreception mechanism is suggested to be based on ferrimagnetic particles within the upper beak innervated by the ophthalmic branch of the trigeminal nerve (V1)^11–16^, and which seem to provide magnetic information for a navigational map^17,18^ This mechanism is consistent with the observation that birds pre-exposed to a brief but strong magnetic pulse (as a tool to perturb a magnetic particle-based magnetoreceptor) had shifted orientations compared to untreated control birds when tested in Emlen funnels^11,19,20^ or in free flight^21,22^ (but see Karwinkel et al. 2022^23^). The observation that pulse effects were restricted to experienced migrants which had already successfully finished at least one migratory journey has led to the notion that adults use magnetic-particle based receptors to acquire magnetic map information. Further evidence in support of the so-called magnetic map theory has come from virtual magnetic displacement studies, where animals tested under magnetic field parameters mimicking a displacement site re-oriented as if they had been physically displaced to that site^24,18^.

From ablation studies, V1 has been identified as necessary for conveying magnetic map information in migratory songbirds^17,18^. In addition, behavioral molecular mapping has shown that birds exposed to a strongly changing magnetic field stimulus display significantly increased expression levels of immediate early genes in subcompartments of the principle and spinal sensory trigeminal brainstem nuclei which receive V1 input^25–27^ but see Kishkinev et al. 2016^28^. The magnetically activated neurons in the principal sensory trigeminal brainstem nucleus were recently shown to define a morphologically distinct neuronal subpopulation, likely to form the origin of a neuronal processing stream exclusively dedicated to transmitting trigeminally perceived magnetic information to higher telencephalic integration centers^29^.

Given that magnetic map information is conveyed by V1 but misinterpreted after magnetic pulsing, it is sensible to postulate that magnetic particles form the basis of trigeminal magnetoreception. With V1 responsible for sensory innervation of the upper beak, iron-rich structures found in the upper beak of homing pigeons and songbirds^12–16^ (but see Winklhofer & Kirschvink, 2008^30^ were considered to represent the long-sought trigeminal magnetic sensor, particularly since the structures were found to contain magnetite nanocrystals in large numbers^31,32^ and suggested to colocalize with nervous tissue^12,13^. Critically, however, the association between iron-rich structures and nervous tissue turned out irreproducible in independent follow-up studies, who found iron-rich structures not associated with nervous tissue but highly colocalized with cells presenting MHC class II, i.e. probably macrophages^33–35^.

**Figure 1:**
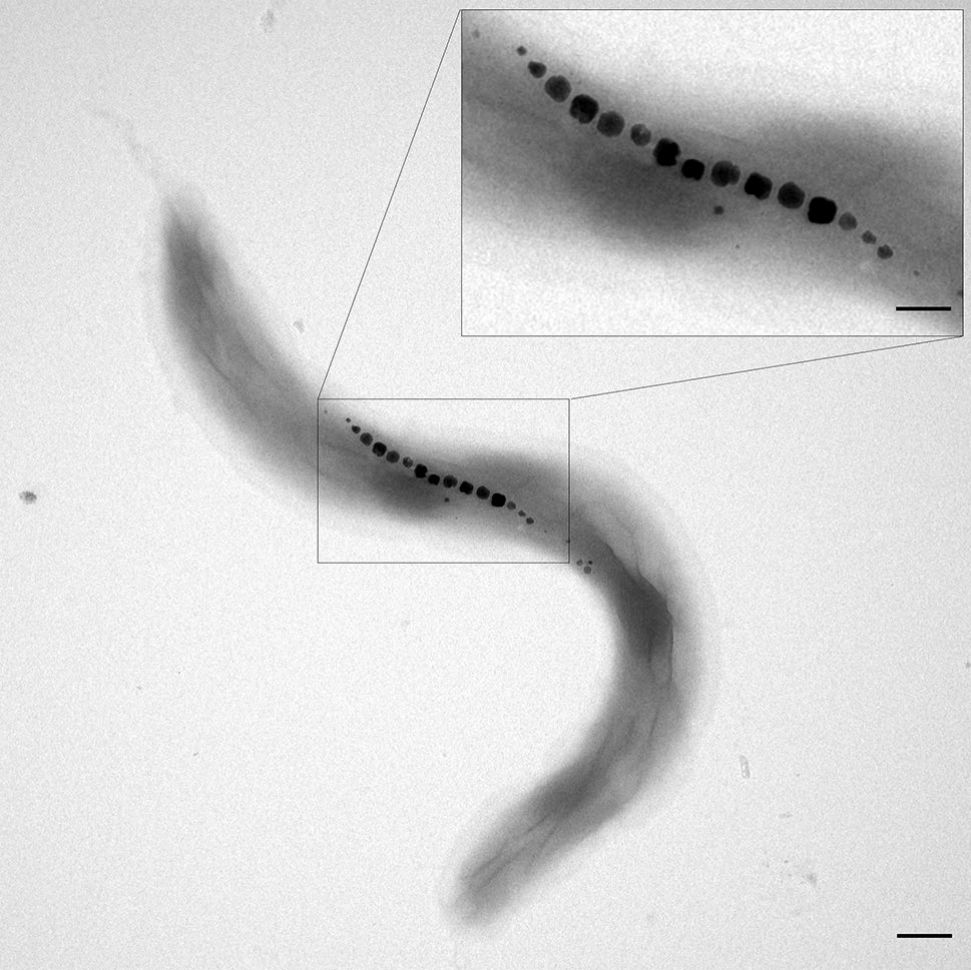
Transmission electron micrograph of *Magnetospirillum magnetotacticum*. In this typical cell, 15 magnetite crystals (electron dense particles) are arranged in a single-stranded magnetosome chain of ca. 500 nm length. Each crystal is surrounded by a membrane vesicle (not visible without membrane stain), which limits the crystal size to ca. 50 nm in this species and is responsible for the apparent gaps between the magnetosomes. Smaller magnetite crystals at the chain ends have not reached their final size and indicate the bidirectional growth of the chain. Scale bar 200 nm, inset scale bar 100 nm.

The majority of these studies screened tissue sections for non-hemin iron-rich structures as possible hints towards magnetosensory structures, using the Prussian blue (PB) staining technique. However, upon closer comparison of earlier PB studies on the upper beak of birds, we realized potential methodological pitfalls, which might have been the cause for the contradictory results of previous studies:

1. Fleissner et al. (2003)^13^ and Treiber et al. (2012, 2013)^33,34^ relied on antibodies against generic neuronal markers such as neurofilaments to find possible colocalizations of PB and nervous tissue. It is not clear if this approach is suitable to also label free nerve endings, where primary sensory receptors are expected to occur.
2. Apart from false negatives, such a generic labelling approach may also yield false positives in the form of PB positive sites colocalizing with nerves of the autonomous system, e.g., for regulation of blood vessels.
3. None of the previous works used a suitable positive control for intracellular magnetite. Although the PB method detects ultrafine iron oxide nanoparticles (< 10 nm) in tissue when present as dense accumulations measuring several hundreds of nm in diameter^31,36^, such nanoparticulate structures are far from representing an optimized solution for realizing a sensor that should be capable of detecting the small magnetic field differences which were implied from the magnetic map experiments mentioned above. For instance, cuticulosomes, which are iron-rich vesicles found in the cuticular plate of hair cells in the Avian inner ear^36–38^, are so weakly magnetic that they would not even qualify for a compass sense to begin with^39^. In contrast, magnetite crystals with particle-sizes between 40 and 100 nm have superior magnetic properties, forming single-domain magnets, which makes them much more suitable as magnetic field sensitive structures^40^, as can be best seen in the example of magnetotactic bacteria (Fig. 1).

These microorganisms biomineralize chains of membrane-enclosed sub-100 nm magnetite crystals, termed magnetosomes, which impart a permanent magnetic dipole moment to the bacterial cell body and keep it thus aligned with the magnetic field^41^. Notably, similar crystals were found in structural magnetoreceptor candidates associated with trigeminally innervated regions of the olfactory epithelium of rainbow trout, using reflectance confocal laser scanning microscopy for screening in combination with electron microscopy or magnetic scanning probe microscopy for validation of iron chemistry or of magnetic properties, respectively^42,43^. In contrast to these methods sensitive to physical properties of single-domain magnetite, it is not known if the PB method is capable of detecting just a dozen of magnetosome-like crystals, which suffice for a magnetoreceptor according to theoretical models^40^. We therefore embarked on a reexamination of the topic, where we studied beak tissues of a night-migratory songbird, the Eurasian blackcap (*Sylvia atricapilla*), in which we specifically labelled V1 fiber terminals within the beak using neuronal tract tracing. As in previous studies^12–15,33,34^, we used the PB technique, for the lack of a better histochemical stain with specificity for the magnetic iron compounds magnetite (or its oxidized form, maghemite)—two components which we would ultimately expect to find in a magnetic-particle based magnetoreceptor structure. None of the previous studies applied the modified PB protocol with diaminobenzidine (DAB) post-enhancement of PB^44–46^ since DAB was already used as chromogen in immunohistochemistry, which is why we did without the modified protocol, too.

To approach point 3) also independently, we applied the PB technique to the olfactory epithelium of rainbow trout, where we expect to find candidate magnetoreceptor structures^42,43^, however in sparse occurrence, and therefore, for ground-truthing, to cells of a magnetotactic bacterium (*Magnetospirillum magnetotacticum* strain MS-1, see Fig. 1), where we know with certainty of the occurrence of intracellular iron-rich target structures.

Since the PB technique is essential for our study, we commence with a brief summary of the chemical reactions involved in PB staining, followed by theoretical considerations exploring the potential sensitivity of the PB stain in relation to various iron oxide minerals found in tissues.

### The Prussian blue staining reactions

The PB stain for ferric ion was introduced by Perls in 1867^47^ and has since been employed widely in biology and pathology. Despite variations in the protocol, all ferric iron staining kits use the hexacyanoferrate (II) anion (“ferrocyanide”), ${\left[{\mathrm{Fe}}^{\mathrm{II}}{\left(\mathrm{CN}\right)}_{6}\right]}^{4-}$, as reagent to bind free Fe^3+^ in the form a blue pigment referred to as a Prussian blue. The reagent is applied in acidic solution, typically hydrochloric acid (HCl) solution, to liberate Fe^3+^ from its bound forms in the tissue, because ferric iron requires pH < 2 to exist as free Fe^3+^ ion (see Pourbaix diagram for the iron-water system in Delahay et al. 1950)^48^; a pH value of 2 corresponds to 10 mM HCl solution or approx. 0.04 wt% HCl. When a crystalline iron oxide is present, e.g., Fe_2_O_3_, the low pH also affords the protons to dissolve the oxide, i.e.,

1 $${\text{Fe}}_{2} {\text{O}}_{3} + { }6{\text{ H}}^{ + } { } - > 2{\text{ Fe}}^{3 + } + { }3{\text{H}}_{2} {\text{O}}$$

Thus, unlike a conventional histochemical stain, which would label a target molecule directly, the PB staining technique requires the (acidic) dissolution of the target compound to then label the liberated target ion. Depending on the mobility of the free Fe^3+^, the PB stain may not mark the original site of the target compound, but rather its diffusion trace. Depending on the ease with which the target compound can be dissolved, it may also disappear altogether. In any case, the target compound will be irreversibly altered.

The actual PB staining reaction is thought to occur in two steps, with the following reaction proceeding first:

2 $${\text{Fe}}^{3 + } + \left[ {{\text{Fe}}^{{{\text{II}}}} \left( {{\text{CN}}} \right)_{6} } \right]^{4 - } + {\text{K}}^{ + } { } - > {\text{KFe}}^{{{\text{III}}}} \left[ {{\text{Fe}}^{{{\text{II}}}} \left( {{\text{CN}}} \right)_{6} } \right]$$

where the potassium ions are delivered with the dissolved reagent, ${\text{K}}_{4} \left[ {{\text{Fe}}^{{{\text{II}}}} \left( {{\text{CN}}} \right)_{6} } \right]$. The product in Eq. (2) is referred to as (water-)soluble PB^49^. In the presence of excess Fe^3+^, insoluble PB precipitates according to:

3 $$4{\text{Fe}}^{3 + } + 3\left[ {{\text{Fe}}^{{{\text{II}}}} \left( {{\text{CN}}} \right)_{6} } \right]^{4 - } { } + { }x{\text{ H}}_{2} {\text{O }} - > {\text{Fe}}_{4}^{{{\text{III}}}} \left[ {{\text{Fe}}^{{{\text{II}}}} \left( {{\text{CN}}} \right)_{6} } \right]_{3} \cdot x{\text{ H}}_{2} {\text{O}}$$

with variable amounts *x* (14 … 16) of unbound water of crystallization in the crystal structure of insoluble PB^50^ (for structure visualization see also^51,52^ Combining Eq. (1) with (3), the complete reaction writes

4 $$12{\text{ H}}^{ + } + { }2{\text{ Fe}}_{2} {\text{O}}_{3} + 3{ }\left[ {{\text{Fe}}^{{{\text{II}}}} \left( {{\text{CN}}} \right)_{6} } \right]^{4 - } + \left( {x - 6} \right){\text{H}}_{2} {\text{O }} - > {\text{Fe}}_{4}^{{{\text{III}}}} \left[ {{\text{Fe}}^{{{\text{II}}}} \left( {{\text{CN}}} \right)_{6} } \right]_{3} \cdot x{\text{ H}}_{2} {\text{O}}$$

Some of the hexacyanoferrate on the left of Eqs. (3 and 4) is derived from the soluble PB forming first (Eq. 2), so that also mixed-phase crystals containing both soluble and insoluble PB can be expected. Either way, all these forms of PB containing iron in mixed valence have blue color. However, after incubation of the tissue with the PB reagent, at least one rinsing step with distilled water ensues, which does not affect the insoluble forms of PB but is likely to wash away the soluble form of PB forming first, which then diminishes the sensitivity and accuracy with which a cellular target can be marked with the technique^53^.

### Theoretical considerations on sensitivity of Prussian blue staining

Despite the vagaries with soluble PB, we here point out that the PB reaction in principle can enhance the apparent amount of iron present. To understand the multiplication effect, it is necessary to compare the volumetric concentration of ferric iron in the iron source material with that in the PB product (Table 1). Strikingly, both soluble and insoluble PB have approximately ten times lower concentrations in ferric iron compared to the possible iron oxide source mineral. This means that the ferric iron liberated from a completely dissolved iron-oxide crystal of initial volume *v*_0_ is equivalent to a PB volume of approximately 10 times *v*_0_ (with reagent ad libitum), hence the enhancement.

**Table 1:** Ferric iron content in Prussian blue in relation to other iron-oxide minerals and iron-oxyhydroxides found in tissues, as calculated from chemical formulae and volumetric mass density.

Compound	Chemical formula	Density [g/cm3]	Fe^III^ conc [mol/cm3]
Prussian blue (soluble)^a^ Prussian blue (insoluble)^b^	KFe^III^[Fe^II^(CN)_6_] Fe^III^_4_[Fe^II^(CN)_6_]_3_ *x* H_2_O (*x* = 14–16)	1.45 1.72–1.78	4.71 6.35
Magnetite^c^	Fe_3_O_4_ Fe^III^_2_O_3_ Fe^II^O	5.21	45.0 (total Fe: 67.5)
Maghemite^d^	γ-Fe^III^_2_O_3_ Fe^III^_2_O_3_ Fe^III^_2/3_O	4.80	61.5
Hematite^e^	α-Fe^III^_2_O_3_	5.27	66.0
Ferrihydrite^f,g^	Fe^III^_8.2_O_8.5_ (OH)_7.4_ Fe^III^_10_O_14_(OH)_2_	4.3 4.9	47.0 60.0

^a^Keggin & Miles 1936^49^, ^b^Buser et al. 1977^50^;

^c,d^Magnetite and its fully oxidized form, maghemite, are strongly ferrimagnetic minerals. The oxidation of magnetite to maghemite produces one vacancy at every third of the former iron(II) sites in the lattice, hence the lower density compared to magnetite. To emphasize the relationship between maghemite and magnetite, the chemical formula of maghemite can also be written as Fe^III^_2_O_3_ Fe^III^_2/3_O or in sum, Fe_8/3_O_4_.

^e,f,g^Hematite and ferrihydrite (for structural details see Michel et al. 2007, 2010^54,55^) are weakly ferrimagnetic minerals. In the biological iron storage protein ferritin, iron is typically stored in the form of a nanocrystalline core of ferrihydrite or hematite, sometimes of a magnetite-like phase (magnetite or maghemite), at least in human brains^56^. Magnetic data of horse spleen ferritin can be explained by a combination of ferrihydrite and a variable but small amount of strongly magnetic phase like magnetite or maghemite^57^.

Next, we ask if the PB method is theoretically sensitive enough to mark a chain of magnetosomes (Fig. 1) such that it can be visualized in a normal wide-field, transmitted-light micrograph taken with a high numerical aperture objective. We assume that all magnetite in a volume *v*_0_ can be dissolved completely. We then compare that volume with known cellular iron-containing structures that were labelled with the PB technique in tissue sections and verified independently with transmission electron microscopy in unstained ultrathin sections. A good example here are cuticulosomes in hair cells of birds, iron-rich structures with diameter of 0.4 µm, densely packed with ferritin nanoparticles^36,37^. Assuming a packing density of 0.5, a ferritin protein shell diameter of 12.5 nm and a mineral core containing 1000–3000 ferric iron atoms (typical iron load in human-liver or horse-spleen ferritin^58^), we obtain the total number of Fe^3+^ ions in a cuticulosome as 1.6 − 4.9 × 10^7^. A typical magnetosome chain in a magnetotactic spirillum consists of 15 magnetite crystals (Fig. 1), with 45 nm edge length each, amounting to an equivalent of 3.7 × 10^7^ Fe^3+^ ions. Judging from this close numerical agreement in ferric iron loads between a magnetosome chain and a cuticulosome, the PB technique is deemed capable of resolving a magnetosome chain under normal transmitted light, which without staining is short of impossible unless special optical contrast enhancement techniques are used. However, there are two important unknowns that remain to be constrained from experiments. First, while magnetite nanocrystals are dissolved completely within 5 min of exposure to a 4% HCl solution^13^, it may take significantly longer to dissolve a 50 nm sized crystal, and yet longer when the crystal is enveloped by a membrane vesicle. Second, it is not known if the acid treatment is sufficient to perforate membranes to a point where the PB reagent, hexacyanoferrate(II), can enter the iron source region. With its large charge and radius, hexacyanoferrate(II) is too lipophobic to permeate a lipid bilayer membrane by passive diffusion. To enter, it requires pores of several nm in diameter to enter, as suggested by exclusion of potassium hexacyanoferrate(III) by sub-2 nm pores in a hydrophobic model membrane^59^. We address these questions by applying the PB technique to whole cells of magnetotactic bacteria.

## Results

### Prussian blue staining products do not colocalize with V1 in beak

**Figure 2:**
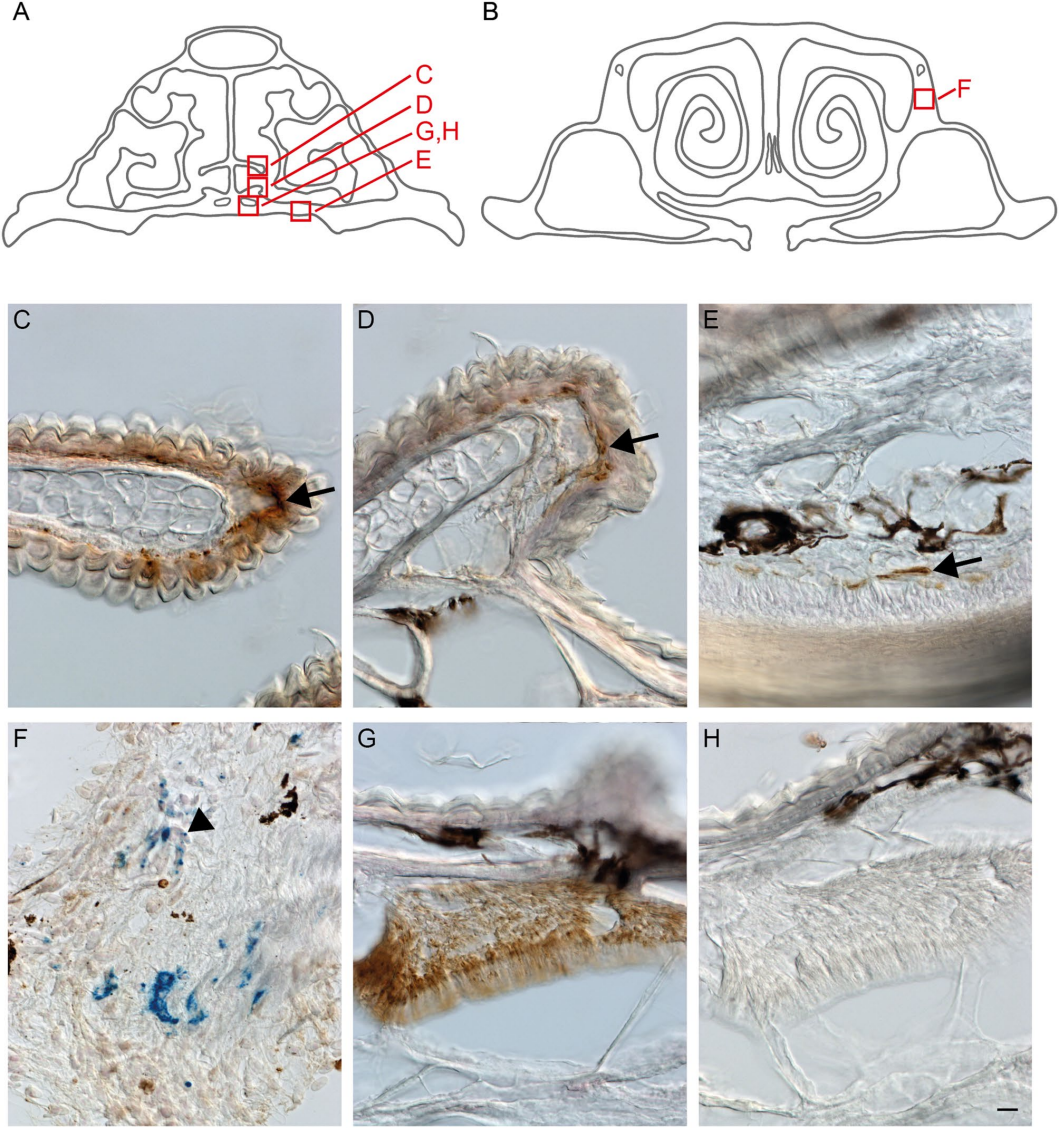
Cholera toxin B subunit labelled fiber terminals of the ophthalmic subbranch of the trigeminal nerve (V1) and Prussian blue labelled structures within the upper beak of Eurasian blackcaps. Upper beaks of Eurasian blackcaps were cut in a frontal plane and labelled with the neuronal tracer Cholera toxin B subunit (brown), Prussian blue (blue) and the nuclear marker nuclear fast red (magenta). (**A**, **B**) The location of the regions of interest shown in C to H are indicated by red boxes in schematic representations of frontal beak slices at a rostral (**A**) and caudal (**B**) level. Fiber terminals of V1 (indicated by arrows) are located (**C**) in outgrowths of the nasal septum, (**D**) in the ventral subepidermal layer, (**D**) in the bulges of the ventral part of the upper beak, and (**E**) in the ventral subepidermal layer at intermediate beak levels. (**F**) Representative image of PB labelled structures. Arrowheads indicate PB-labelled structures colocalized with nuclear fast red. (**G**) CtB labelling in V1 is shown as positive control and (**H**) the same structure when the primary antibody was omitted as negative control. Black structures in the beak slices represent endogenous pigment of the upper beak. Scale bar: H (for C-H), 10 µm.

To investigate if iron-containing structures are located in close proximity to nervous structures in the upper beak, nerve fiber terminals of V1 and PB labelled structures were analyzed for possible colocalization, using the night-migratory songbird Eurasian blackcap as a model species (Fig. 2A-H). V1 nerve fiber terminals were mainly located in outgrowths of the nasal septum of the upper beak (Fig. 2A,C), in bulges in the ventral part of the upper beak (Fig. 2A, D), and in the ventral subepidermis of the upper beak (Fig. 2A,E). We found no PB positive structures located in close proximity to these nerve fiber terminals (Fig. 2C-F). Punctiform structures of PB appeared partially nucleated shown by a colocalization of PB and the nucleic acid marker nuclear fast red (NFR; Fig. 2B,F).

**Figure 3:**
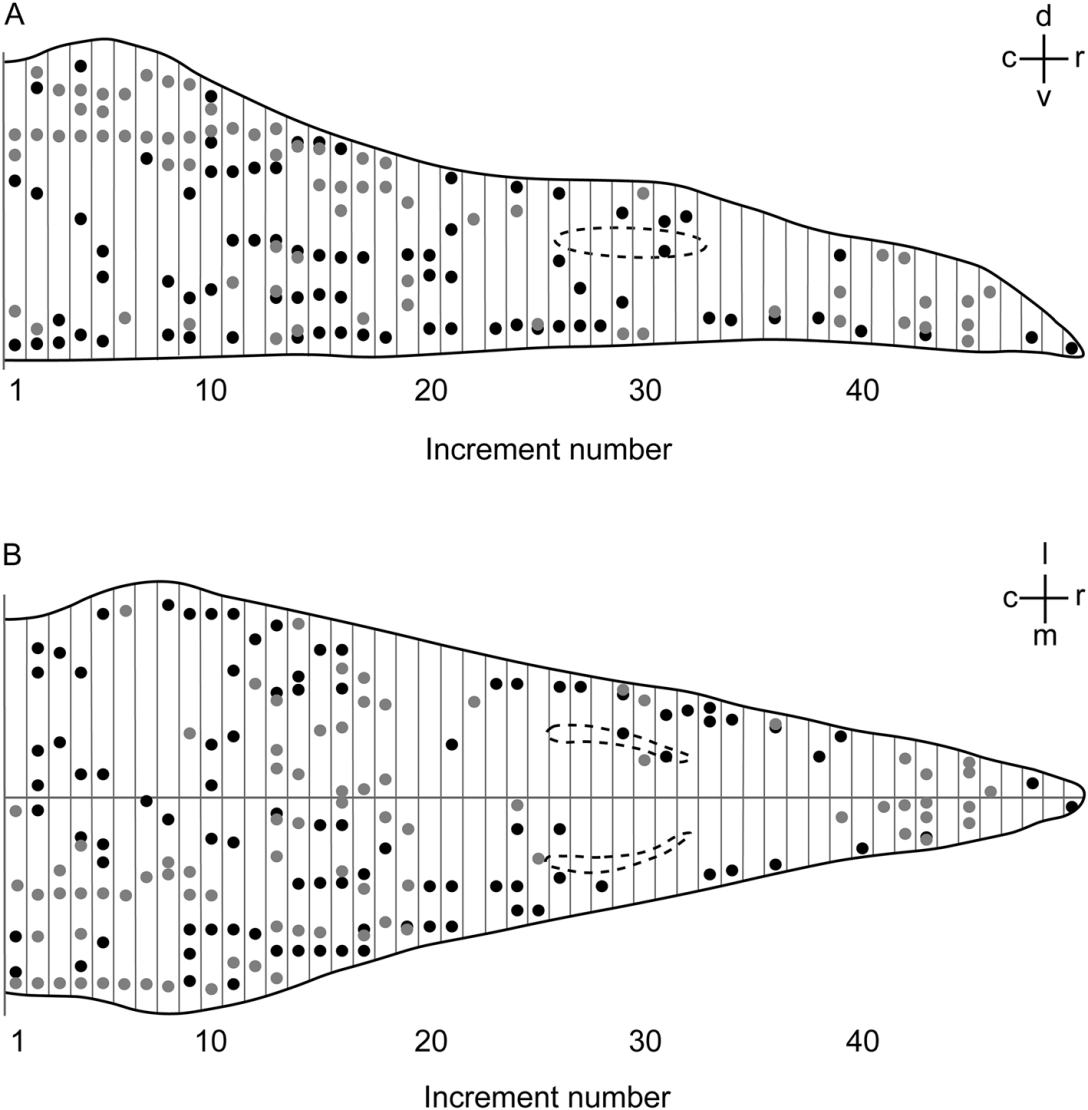
Distribution of Prussian blue (PB) positive sites in the upper beak of Eurasian blackcaps. PB positive sites are shown along the (**A**) dorso-ventral axis and (**B**) medio-lateral axis of the upper beak in 50 250 µm-increments from caudal to rostral. Each dot represents PB positive sites. Black and grey dots represent two different individuals. Dashed lines represent the location of the nostrils. Abbreviations: c, caudal; d, dorsal; l, lateral; m, medial; r, rostral; v ventral.

In general, the overall spatial distribution of PB positive structures in the upper beak strongly varied between the two individuals analysed and did not appear to follow an ordered pattern (Fig. 3). These findings were overall consistent with previous studies^33–35^), which also failed to observe the six distinct PB sites reported in the original study^13,14^. The only approximate systematic tendency we noticed was a difference between caudal and rostral areas in terms of PB site clustering and anatomical locations: in caudal regions of the beak, PB positive structures were mainly located in the dorso-lateral parts and formed larger clusters (Figs. 2B,F and 3), while PB sites in more rostral parts were sparse and occurred in lateral and ventro-medial parts (Fig. 3).

### Prussian blue labels trapped particles in olfactory epithelium of fish

**Figure 4:**
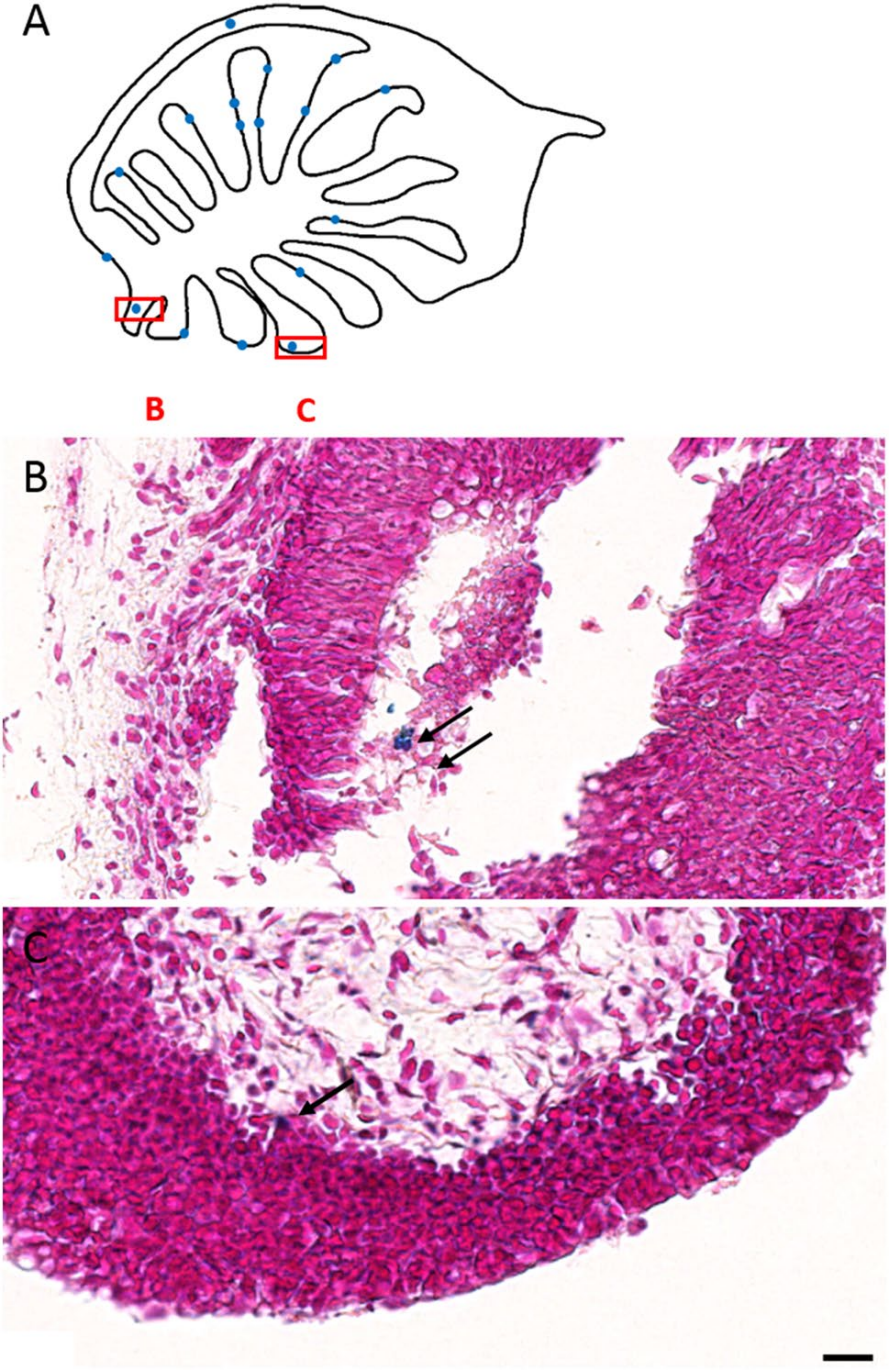
Prussian Blue (PB) stained olfactory rosette from rainbow trout, counterstained with nuclear fast red. (**A**) Camera lucida view of a horizontal section of the olfactory capsule, showing lamella with olfactory epithelium folds branching off the central raphe. PB positive sites, marked as blue dots, are by and large located at the luminal side of the olfactory epithelium and most likely represent contamination from external iron-rich particles sticking to the epithelium. (**B**) An example of a large PB stained feature (arrow) between epithelial cells, in close proximity to the lumen, and a second smaller one located in the lumen above. (**C**) A PB positive site at the basal layer of the epithelium, close to the apex of a lamella fold, where candidate magnetoreceptor structures have been identified earlier^42,43,61^. Upon closer look, however, this seemingly promising site is rather defocused compared to the surrounding tissue and turns out to lie above the tissue, so that it therefore most likely represents an external iron-rich particle entrained in the sectioning process. Scale bars 20 μm.

Having found no PB positive sites associated with V1 in the upper beak of Eurasian blackcaps, we next applied the PB protocol to olfactory rosettes of rainbow trout, where candidate magnetoreceptor structures have been reported earlier as cells containing highly reflective particles^42^ with magnetic properties^43,60,61^. According to the tracings of Walker et al. (1997)^42^ with the lipophilic dye DiI, some processes of the superficial ophthalmic branch of the trigeminal nerve (referred to as rosV) enter into the olfactory lamellae from their tips down to the lamina propria, where candidate magnetoreceptor structures have been consistently found, albeit in low numbers. In our PB-treated sections of olfactory rosettes (Fig. 4), however, we did not observe any meaningful PB staining patterns. The vast majority of PB sites were found at the luminal side of the epithelium, which at the same time forms the interface with the aqueous environment through the nasal openings. It is thus well conceivable that iron-rich fine-dust particles suspended in water flowing through the olfactory organ were trapped at the external surface of the epithelium. Therefore, no matter how much precautions regarding clean lab environments are taken^62,63^, external particles already incorporated in vivo remain a constant source of concern when dealing with olfactory, gustatory, and other sensory epithelia bathed in environmental water, such as lateral line canals. This also needs to be borne in mind when assessing non-luminal PB sites, such as the one shown in Fig. 4C. Simply by varying the focal plane under the microscope, we found this particular PB site (and similar ones in other sections) to be in focus in a plane where the tissue is not yet in focus, which means that the PB site is not situated in the tissue. These non-luminal PB sites therefore are likely derived from entrained external particles, which were sheared across during the sectioning process.

With regard to the NFR counterstain, we often found blueish color seams around NFR stained nuclei, which may be mistaken as PB stains. These color artifacts are a clear manifestation of chromatic aberration, which despite apochromatic objectives, often emerge when imaging curved objects with slightly higher density compared to their surroundings, such as nuclei against cytoplasmic background. The curvatures may act as additional microlenses in the transmitted light path.

### Prussian Blue does not label magnetosomes in bacteria cells

**Figure 5:**
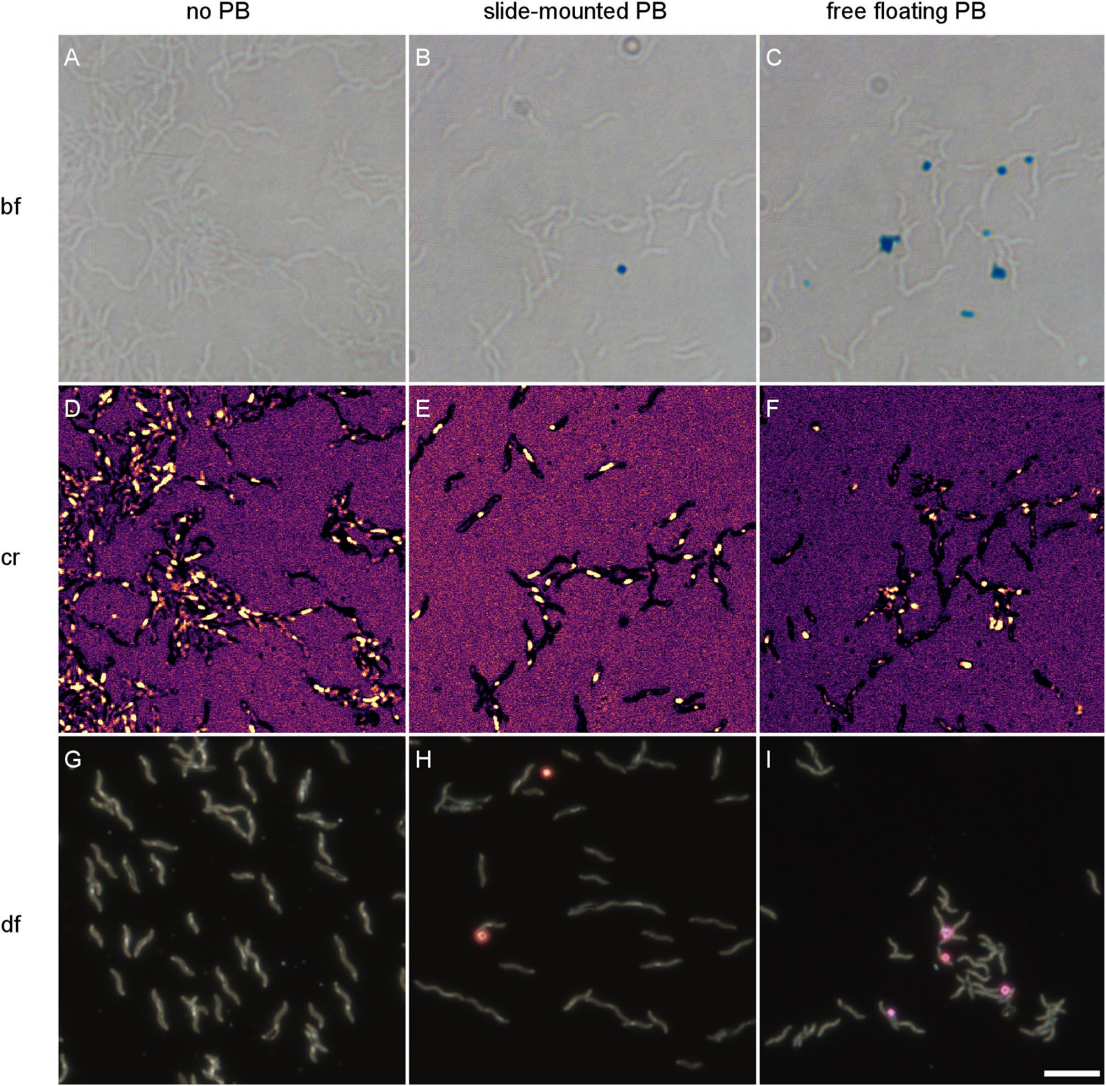
Prussian Blue (PB) method does not stain intracellular magnetosome chains in *Magnetospirillum magnetotacticum.* (**A**, **B**, **C**) transmitted-light bright-field (bf) images of A: untreated cells, B: slide-mounted cells treated with PB protocol, C: free floating cells treated with PB and placed on microscopy slide afterwards. Some of the suspension was placed on a copper grid for further analysis with transmission electron microscopy (Figs. 6 and 7). PB reaction products can be clearly recognized by their blue color (**B**, **C**), but are definitely extracellular in the slide-mounted preparation (**B**), with a clear distance to the cell bodies. In the free-floating cell preparation (**C**), the PB reaction products appear closer to the cell, which likely represents a centrifugation artifact. (**D**, **E**, **F**) Same field of view as in A, B, C, respectively, but imaged with confocal reflectance (cr) at 488 nm, featuring intracellular magnetosome chains as bright reflective objects. Depending on their crystal size and degree of crystallinity, PB reaction products may or may not appear under confocal reflectance (**E**, **F**). (**G**, **H**, **I**) Enhanced dark field (df) images of the same slides as in D, E, F, respectively, albeit with different field of views. Under dark-field contrast magnetosome chains appear as slender white objects, while PB reaction products appear in their complementary color (orange to pink). As in (**B**), the PB reaction products in (**H**) are clearly extracellular. In particular, all magnetosome chains have the same white color as in the untreated sample (**G**), which independently confirms the lack of PB stainability of intracellular magnetosome chains. Scale bar 5 μm.

Having failed to find promising PB sites in Eurasian blackcap beaks and trout olfactory rosettes, we engaged in some ground truthing work on bacteria, where we know that magnetite is present. When applied to whole cells of *M. magnetotacticum* (Fig. 5A-I), the PB method does not label the magnetite chains but instead yields extracellular staining products, which can be clearly seen as blue spots in bright-field transmitted light images (Fig. 5B,C) and as orange or pink spots in enhanced-darkfield imaging, which collects scattered light and thus inverts the color seen in transmitted light (Fig. 5H,I). The magnetosome chains remain in place, as shown in the confocal reflectance images (Fig. 5D-F) as well as in the enhanced darkfield images (Fig. 5G-I). In bright-field transmitted light, the magnetosome chains would appear black, but do not produce enough contrast to be visible. We found very similar results when applying the PB reagent in HCl solution immediately to slide-mounted cells without the prior HCl incubation step intended to prolong the iron leaching time.

**Figure 6:**
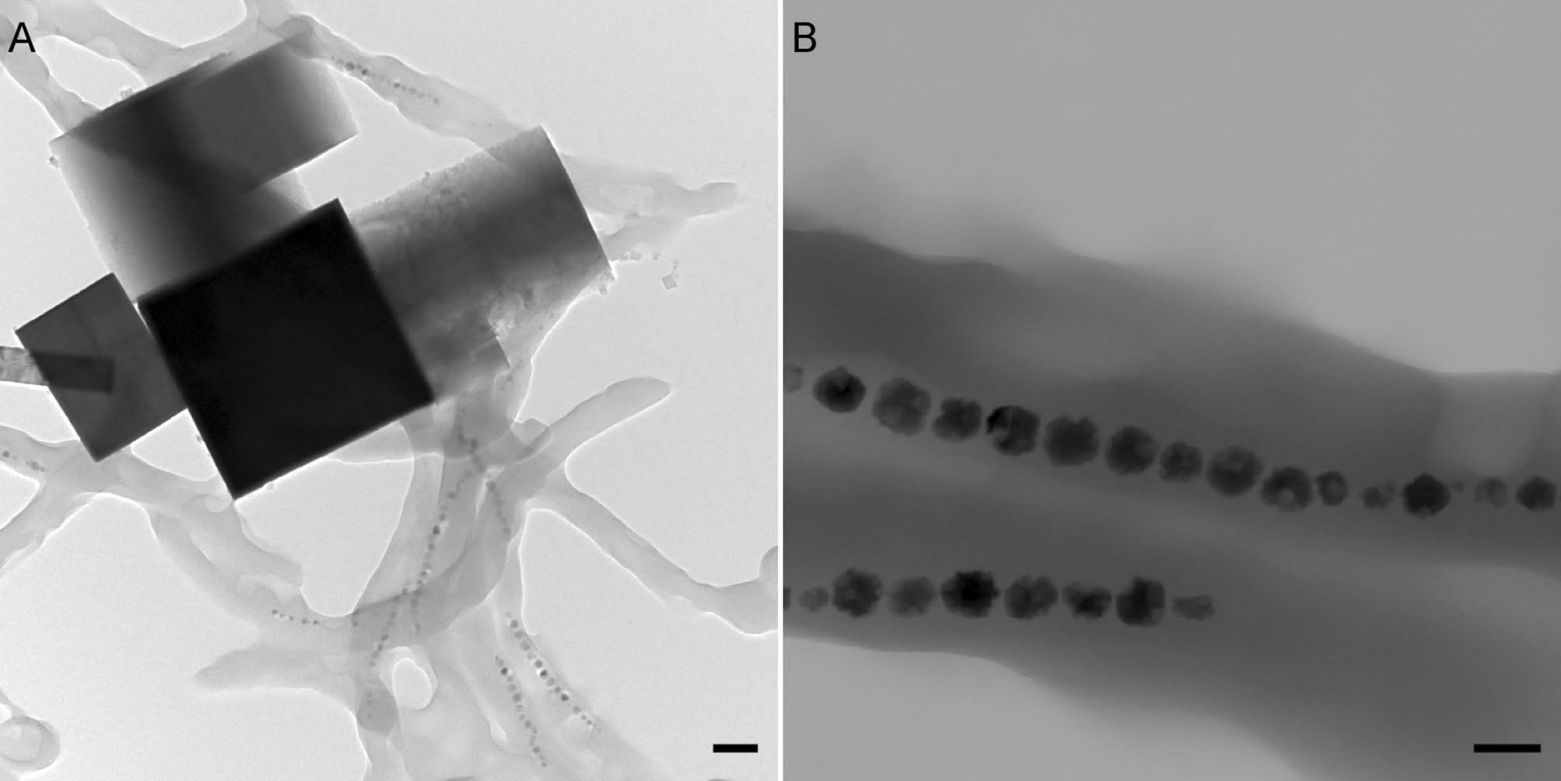
Transmission electron micrographs of cells of *Magnetospirillum magnetotacticum,* after Prussian blue (PB) treatment of free-floating cells. (**A**) The magnetosome chains in the cells are not labelled by PB and remain in place (lower right). The actual PB reaction products are the micrometer-sized box-like structures in the upper left corner of the image, with the electron dense cube in the middle representing an insoluble PB crystal, as determined from its diffraction pattern. (**B**) At higher magnification, the magnetosome chains are still present, but the magnetosome particles appear to have lost their distinct crystal edges and rather have etching pits, which suggests partial dissolution, attributable to the acid treatment (as part of the PB protocol). It can be shown that the visible chains still consist of iron oxide using energy-disperse X-ray scans (see Fig S2). Scale bar 200 nm (**A**) and 50 nm (**B**).

**Figure 7:**
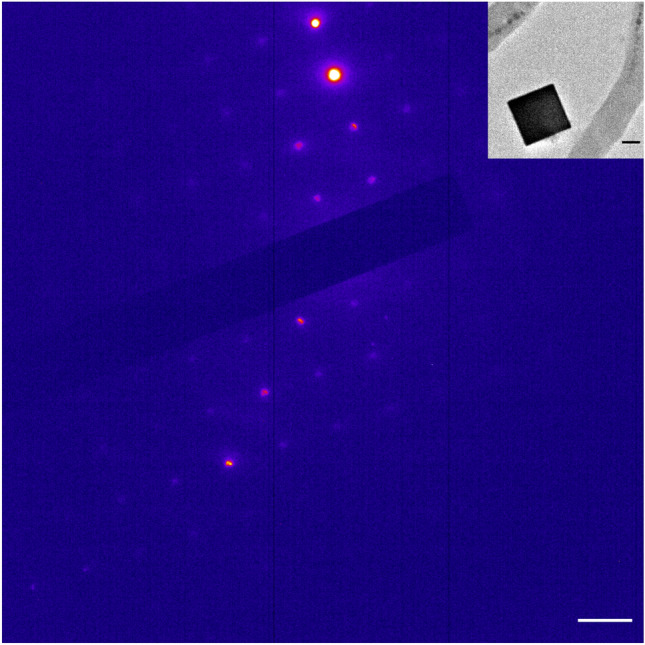
Selected area electron diffraction (SAED) pattern of an insoluble Prussian blue (PB) single crystal (see image in inset), aligned with a four-fold symmetry axis to the electron beam. The diffraction pattern and reciprocal lattice spacing of 0.985 nm^−1^ is consistent with the simple cubic lattice of insoluble PB (space group P m -3 m, lattice constant 1.0166 nm). Scale bar 1 nm^−1^, scale bar inset 100 nm.

The electron dense extracellular crystals with square faces (Fig. 6), can be identified as PB crystals by their characteristic electron diffraction pattern and lattice spacings (Fig. 7). The PB crystals appear to be mixed-phase crystals between soluble and insoluble PB, as indicated by the coexistence of potassium with iron (soluble PB, Fig. S1) and the extra diffraction spots of insoluble PB (Fig. 7). Our observation that PB reaction products are extracellular prompts two questions: (i) Why do magnetosomes defy PB staining and (ii) what is the source of extracellularly PB stained ion?

(i) In cells of magnetotactic bacteria, magnetosomes present, by far, the highest local concentrations of ferric ion (ca. 45 M, see Table 1), which however is chemically locked in the magnetite host crystals and therefore needs to be liberated to be accessible to the PB reagent. The necessary leaching process is afforded by the strong acid solution applied with the PB reagent, which in dead cells (i.e., in absence of active pH homeostasis mechanisms) is sufficient to increase the intracellular proton concentration to the point of dissolving magnetite at least partially. This should leave behind etching pits on magnetosomes, which is supported by our electron micrographs showing irregularities on the surface of the magnetosomes (Fig. 6), similar to the corrosion features observed in fossil magnetosomes extracted from deep-sea and lake sediments undergoing changes in pH-redox conditions^64,65^. However, compared to the small proton, the much larger hexacyanoferrate(II) anion with its significantly higher charge ($z=-4$) is too lipophobic to permeate both intact bacterial cell wall and magnetosome membrane during the incubation time by passive diffusion*.* In fact, in the EDX-based elemental analysis of magnetotactic bacteria subjected to the PB staining procedure on floating cells (SI Fig. S2), the only major elements detected in magnetosomes were iron and oxygen from the original magnetite composition, but oxygen is incompatible with Prussian blue. Therefore, the most likely explanation is that hexacyanoferrate did not enter cells nor magnetosome vesicles.

(ii) There are several possibilities for the source of PB-stained ferric iron in the extracellular space. To exclude the possibility of ferric iron leftovers from the culture medium containing ferric quinate, despite several washing steps of the cells prior to PB treatment, we applied the same procedure to cells of nonmagnetotactic bacteria (*Aromatoleum aromaticum,* strain EbN1^66^). In 20 replicates of EbN1 cells pre-immersed in ferric quinate containing medium, we did not observe any PB staining products (Fig. S3A). In a second series of 20 replicates, where EbN1 cells had not been pre-exposed to ferric quinate, we observed merely a single PB crystal (Fig. S3B), which we ascribe to contamination. Given the low occurrence of PB staining products in our negative controls, we suggest that ferric iron derived from magnetosomes does contribute to extracellular PB staining products in the magnetotactic bacteria samples.

## Discussion

In contrast to the PB study on V1-traced pigeon beaks by Williams and Wild (2001)^12^, we did not find any instance of PB colocalization with V1 in the upper beak of Eurasian blackcaps. This should not be taken as evidence against V1-mediated magnetoreception in this songbird given all the independent lines of evidence for trigeminal-based magnetoreception in night migratory songbirds mentioned earlier. However, our findings strongly suggest the absence of clusters of superparamagnetic magnetite-maghemite nanoparticles in or at V1 fiber terminals in the upper beak of Eurasian blackcaps. Such clusters were identified earlier in the upper beak skin of homing pigeons with the aid of electron microscopy techniques and did produce an intense PB stain in microtome sections^31,32^, regardless of the fact that their spatial association with antibody-labelled neurofilaments^13^ turned out to be irreproducible in an independent follow up study by Treiber et al. (2012)^33^. The absence of colocalization between PB-staining and V1 fiber terminals in Eurasian blackcaps therefore confirms the negative result on homing pigeons by Treiber et al. (2012)^33^, who used antibodies against neuronal cytoskeletal proteins. Likewise, the PB stained structures seen in Fig. 2F resemble the ones in the nucleated cells shown in Treiber et al. (2012)^33^, who proposed macrophages as host cells of PB-stained iron-rich structures, representing hemosiderin deposits, i.e., proteolyzed ferritin aggregates.

PB-stainable iron structures certainly comprise hemosiderin aggregates, but iron sources of external origin need to be considered too. The problem of contamination with externally sourced iron trapped already in vivo is most obvious in the olfactory rosettes of rainbow trout, where we found many instances of PB-positive sites at the lumen-side of the olfactory epithelium (Fig. 4). These PB staining products most likely represent iron-rich nanoparticles that were originally suspended in the water flow through the olfactory capsules. Similarly, the breathing passage of birds and other land animals is exposed to ultrafine dust suspended in the incoming air flow. This would not matter if the dust particles were staying on the surface of the epithelium, where they can be easily distinguished as external contaminants. Worryingly, ultrafine particles deposited on the olfactory mucosa of rats were found 24 h later in the olfactory bulb^67^, which suggests that the particles infiltrate olfactory sensory neurons and diffuse anterogradely in the axoplasm into the brain, even across the synaptic cleft. It therefore comes as no surprise that combustion-derived magnetite nanoparticles have also been detected in the olfactory bulb of humans exposed to heavy air pollution^68^. Despite a vast body of literature on uptake of particulate matter by cells [see reviews by Behzadi et al. 2017^69^, Mosquera et al. 2018^70^, Foroozandeh & Abdul Aziz 2018^71^, Donahue et al. 2019^72^], the precise mechanisms by which uncoated nanoparticles infiltrate peripheral neurons are not known, but given their small size, they may enter, suspended in colloidal form, by way of pinocytosis, an unspecific uptake mechanism of extracellular fluid. In the case of olfactory sensory neurons, we propose that odorant receptor internalization via a clathrin-dependent endocytotis mechanism^73^ can provide an entry portal for nanoparticles, allowing them to slip in with the odorant-bound receptor. Since G-protein coupled receptors (GPCR) are found in other types of cells, too, notably in nociceptive somatosensory terminals (e.g., Basbaum et al. 2009^74^) and on the apical membrane of airway epithelia (e.g., Kreda et al. 2000^75^), clathrin-dependent GPCR-receptor internalization could generally act as a collateral uptake mechanism for dust nanoparticles in the nasal passage. Last, macrophages may take in PB-stainable nanoparticles either directly, because macrophages are known to engulf iron-oxide nanoparticles^76–78^, or indirectly by phagocytosing tissue cells with infiltrated nanoparticles.

In the context of nanoparticle infiltration, we note that Williams & Wild (2001)^12^ detected PB-structures associated with V1 mainly in the bony cavities of rostral concha, which is the first anatomical structure in the nasal passage, and that Treiber et al. (2012)^33^ found approximately half of all PB-stained structures in the respiratory epithelium. To us, this indicates that a non-negligible fraction of PB detectable iron must have been incorporated from an external source when the animal was still alive. Thus, given the apparent ease with which nanoparticles find their way into nerve tissue, the presence of PB stainable magnetic nanoparticles in nerves should be considered with caution. We suggest to also include the olfactory nerve or bulb as control tissue to assess the potential contamination in future studies.

To our surprise, we did not obtain any meaningful PB stainings in the subepithelial layers of trout olfactory rosettes, where a candidate magnetoreceptor structure based on single-domain magnetite have been detected by their pronounced confocal reflectance contrast^42,43,61^. This absence of PB products is consistent with our observations on cells of magnetotactic bacteria, where the PB method failed to label intracellular membrane-enclosed magnetite crystals (Fig. 5B,E,H) but instead produced PB pigments in low numbers and at a clear distance to the cells. It has been speculated repeatedly that a magnetosome chain might be too small to be detectable by the PB stain. On the other hand, our theoretical considerations suggest that the amount of ferric iron present in magnetosomes is sufficient to produce a PB pigment large enough to be visible under the light microscope, provided that magnetite dissolves completely. For the PB technique, the problem with magnetosomes rather is their large size in comparison to nanoparticles and therefore the small surface-to-volume ratio, which limits the dissolution kinetics. Indeed, intracellular membrane-enclosed magnetite crystals appeared to remain largely unaffected, even under hydrochloric acid treatment, except for developing some etching pits at the surface (Fig. 6), with the result of supplying only a fraction of the ferric iron pool stored in magnetosomes. While this mechanism readily explains the low amount of PB pigments observed in the PB-treated slide-mounted cells (Fig. 5B,E,H), it does not account for the second observation that PB products here do not colocalize with the ferric iron source region (allochtonous PB). The PB reagent hexacyanoferrate(II) is too lipophobic to permeate the lipid bilayer membranes by passive diffusion. The two factors—large surface-to/volume ratio of magnetite in magnetosomes and membrane enclosure—severely debilitate the reliability of the PB staining technique in terms of sensitivity and site fidelity, which would also apply to the modified PB technique with DAB post-enhancement of PB reaction products. In contrast, clustered nanoparticles, with crystalline cores below 10 nm, have a large surface-to-volume ratio that allows for efficient dissolution by the acidic carrier liquid of the PB reagent and concomitant formation of a PB reaction product. Strictly speaking, the PB technique here does not exactly mark the original compound either but stains the liberated iron diffusing away from the acid-dissolved source. Due to the smallness of the nanoparticles, this parautochtonous staining would go unnoticed under the light microscope.

From our negative PB results on samples known to contain single-domain magnetite, we suggest that the aforementioned PB studies on the pigeon beak were unable to label magnetosome chains even if present and thus prone to miss out candidate magnetoreceptor structures based on membrane-enclosed single domain magnetite. The only case we are aware of where the PB technique was able to stain single-domain magnetite in a tissue are gills of a bivalve containing endosymbiotic magnetotactic bacteria, where intense PB products were obtained in eukaryotic host cells digesting the endosymbiont^79^. Here it was most likely the advancing lysosomal degradation of the magnetosome membranes which was conducive to the PB staining method. A lipase/protease pretreatment mimicking lysosomal processes may be necessary for detecting membrane-enclosed magnetite crystals with the PB technique, but given the likely side effects on the tissue (disintegration, denaturation), we instead suggest to use an altogether different combination of screening techniques that do not require acid dissolution and instead are sensitive to distinct properties of magnetite crystals, such as confocal reflectance combined with Raman-spectroscopy^80^ and nitrogen-vacancy based magnetometry, which has recently been applied to measuring in situ magnetic properties of cuticulosomes^39^. Once candidate structures are detected, contamination has to be ruled out beyond doubt to establish a *bona fide* anatomical structure for the study of magnetic-particle based magnetoreception. Ironically, the Prussian blue technique may be most useful in magnetoreception research for assessing the level of background contamination with iron-rich fine dust.

## Methods

### Eurasian blackcaps (*Sylvia atricapilla*)

#### Animals and housing

Two male adult Eurasian blackcaps (*Sylvia atricapilla)* were wild-caught using mist nets near the University Oldenburg. The birds were kept as pairs in indoor wire cages (102 × 50 × 40 cm) at the institute´s animal facility at around 21 °C and were exposed to a circannual and circadian light–dark cycle simulating the natural light–dark cycle of Oldenburg. Food and water were provided ad libitum. All animal procedures were approved by the Animal Care and Use Committees of the Lower Saxonian State Office for Consumer Protection and Food Safety (LAVES, Oldenburg, Germany, Az.: 33.19-42,502-04-15/1865; 33.19-42,502-04-20/3492).

#### Neuronal tract tracing

Distal fibre terminals of V1 within the upper beak were visualized by neuronal tract tracing. Birds were anaesthetized with Isoflurane CP® (~ 1–1.5% Vol. dissolved in oxygen; 1 ml/ml; cp-pharma, Burgdorf, Germany). V1 was accessed unilaterally through an incision along the dorsal rim of the orbit and careful retraction of the eyeball and oculomotor muscles. This procedure was identical to previous studies^6,17,18,25–27,29^. Approx. 250 nl of the neuronal tracer substance Cholera toxin subunit B (CtB; 1% in distilled water; C9903, Sigma-Aldrich, St. Louis, MO, USA) was administered by pressure injection into the nerve using a microinjector (WPI-2000, World Precision Instruments, Sarasota, FL, USA) and bevelled glass capillaries. After the injection, all tissues were repositioned and resealed using cyanoacrylate surgical glue (Histoacryl®, BRAUN, Rubi, Spain). For post-surgical analgesia, each bird was administered meloxicam (Metacam®, Boehringer Ingelheim, Ingelheim, Germany), 0.1 ml/kg body weight dissolved in 0.9% sodium chloride (NaCl), intramuscular 24- and 48-h post-surgery. Each bird was given three to six days to recover from the surgery and to let the tracer transport.

#### Immunohistochemistry and Prussian blue staining

Birds were deeply anaesthetized with pentobarbital (Narcoren®, Boehringer Ingelheim, Ingelheim, Germany; 2.5 ml/kg body weight) and transcardially perfused using 0.9% NaCl followed by 4% paraformaldehyde (PFA) dissolved in phosphate buffered saline (PBS; pH 7.4). Beaks were post-fixed in 4% PFA in PBS for 24 h and cryoprotected in 30% D(+)-saccharose dissolved in PBS for at least 48 h. Beaks were cut in 25 µm thick slices in the frontal plane in ten parallel series using a freezing microtome (Leica CM 1860, Wetzlar, Germany) and a polytetrafluoroethylene coated knife (Thermo Fisher Scientific, Waltham, MA, USA). Slices were dried on gelatinized glass slides (Menzel SuperFrost® Plus, Thermo Fisher Scientific, Waltham, MA, USA), and stored at − 20 °C until being subjected to immunohistochemistry.

Each of the parallel series of beak slices were stained in one run. To visualize CtB, slices were washed in Tris-buffered saline (TBS; pH 7.6). Endogenous peroxidases were saturated with 0.3% hydrogen peroxide for 30 min and washed three times for 5 min each. Unspecific binding sites were blocked with 10% normal donkey serum (NDS; Antibodies-online, Aachen, Germany) dissolved in TBS containing 0.3% Triton-X100 (TBS-T; Carl Roth, Karlsruhe, Germany) for 30 min. Slices were incubated with a polyclonal rabbit anti-CtB antibody (working dilution 1:1000 in 5% NDS in TBS-T; Sigma-Aldrich, St. Louis, MO, USA, cat. #C3062, lot. #045M4864V, RRID: AB_258833) overnight at 4 °C. After washing three times 10 min each in TBS, slices were incubated with a biotinylated antibody (working dilution 1:200 in TBS-T; PK-6101, Vector Laboratories, Burlingame, CA, USA) for 120 min followed by three washing steps in TBS-T for 10 min each and incubation in an avidin-coupled peroxidase complex (according to the manufacturer’s instructions, PK-6101, Vector Laboratories, Burlingame, CA, USA) for 60 min. Two washing steps in TBS for 5 min each followed. The immunosignal was visualized using a 3′3-diamino-benzidine (DAB) reaction according to the manufacturer’s instructions (SK-4105, Vector Laboratories, Burlingame, CA, USA).

For Prussian blue staining, after three washing steps of 5 min each in distilled water, the slices were incubated in 5% potassium hexacyanoferrate and 5% hydrochloric acid for 20 min according to the manufacturer’s instructions (Hematognost Fe®, 112,084, Sigma-Aldrich, St. Louis, MO, USA). After another three washing steps of 5 min each with distilled water, slices were counterstained with 0.1% nuclear fast red (working dilution 1:12 in distilled water). Beak slices were rinsed in distilled water, dehydrated in a graded alcohol series (70% ethanol, 96% ethanol, isopropanol, twice xylene) and cover-slipped with Eukitt (Sigma-Aldrich, St. Louis, MO, USA). Negative controls were done on parallel beak slices by omitting the primary antibody. Slices were imaged using light microscopy (Nikon Eclipse Ni-Ei, Nikon, Minato, Tokyo, Japan) using an 40 × CFI Plan-Fluor, 0.75 NA objective. Contrast was adjusted with identical settings using ImageJ^81^.

#### Antibody characterization

The used anti-Cholera toxin subunit B (CtB) antibody (Sigma-Aldrich, St. Louis, MO, USA, Cat. #C3062, Lot. #045M4864V, RRID: AB_258833, working dilution: 1:1000) is a polyclonal antibody of the host species rabbit. It was obtained after immunization from a virulent strain (CTXΦ^+^) of *Vibrio cholerae*. CtB (KEGG Entry K10929) is physically absent in any animal unless harboring toxigenic *Vibrio cholerae*, which we can rule out here, thus making any unspecific binding highly unlikely.

#### Analysis of Prussian blue positive structures

To visualize the location of Prussian blue positive structures in the upper beak of Eurasian blackcaps, the Prussian blue labelled structures were identified in 50 stained slices against CtB, Prussian blue, and Nuclear Fast Red in 250 µm increments. The identified locations were transferred to a schematic drawing outlining the upper beak at a dorso-ventral axis and medio-lateral axis.

### Rainbow trout (*Oncorhynchus mykiss*)

#### Animals and housing

Three juvenile rainbow trout (*Oncorhynchus mykiss*) were used for this study. Fish were obtained from a local breeder (Fischfarm Schubert, Wildeshausen, Germany) and kept in a 150 l aquarium at 12 °C under a 14:10 h light: dark cycle. Fish were fed once daily with commercial fish food pellets. All animal procedures were approved by the Animal Care and Use Committees of the Lower Saxonian State Office for Consumer Protection and Food Safety (LAVES, Oldenburg, Germany, Az.: 33.19-42,502-04-17/2450). All experiments were carried out in accordance with the approved guidelines.

#### Prussian blue staining of trout olfactory epithelium

Rainbow trout were deeply anaesthetized with clove oil, transcardially perfused with 0.9% NaCl followed by 4% PFA in PBS (pH 7.4) and decapitated. The heads were post-fixed by immersion in 4% PFA for 24 h. After thorough washing with PBS (pH 7.4), the olfactory rosettes were dissected using titanium alloyed forceps and micro spring scissors. The nostrils were covered with PBS (pH 7.4) to prevent them from drying out during this process. The isolated rosettes were cryoprotected with 30% D(+)-saccharose in PBS for 72 h at 4 °C, embedded in Tissue-Tek® O.C.T.™ Compound (Sakura Finetek Europe B.V., Alphen aan den Rijn, Netherlands) and horizontally sectioned (20 µm) using a cryostat (Leica CM 1860, Wetzlar, Germany) with polytetrafluoroethylene-coated broadband blades (Thermo Fisher Scientific, Waltham, MA, USA). The sections were mounted onto SuperFrost Plus™ GOLD glass slides (Thermo Fisher Scientific, Waltham, MA, USA) and stored at − 20 °C until subjected to the staining procedure.

For PB staining, the frozen sections were placed on a heating plate (37 °C) for 2 h. The rest of the procedure was conducted as per the manufacturer´s instructions (Hematognost Fe®, 112,084, Sigma-Aldrich, St. Louis, MO, USA), as already described in Section 2.1.3. above. Subsequently, the sections were counterstained with 0.1% nuclear fast red (1:3 in distilled water), rinsed with Milli-Q water and dehydrated in ascending alcohol series. Finally, the sections were cover-slipped with Eukitt (Sigma-Aldrich, St. Louis, MO, USA) and imaged under transmitted light (Zeiss Axio Scan.Z1, Oberkochen, Germany), using a 10 × Plan-Apochromat, 0.45 NA objective.

### Bacteria

#### Cultivation of magnetotactic bacteria

Cells of *Magnetospirillum magnetotacticum*, strain MS-1 (Alphaproteobacteria, No. 3856, DSMZ, Braunschweig, Germany) were cultivated at 30 °C under microaerophilic conditions in Magnetospirillum culture medium (No. 380, DSMZ, Braunschweig, Germany) containing succinate and tartrate as main electron donors and ferric quinate as iron source; sodium-thioglycolate, the reducing agent from the original recipe, was replaced by Na_2_S. After cultivation, cells were stored at 8 °C before harvesting magnetically enriched samples of cells. For this purpose, magnetic cells were separated with a NdFeB magnet and centrifuged. The pellet was re-suspended in 4% PFA, then washed in PBS and distilled water to obtain a magnetically enriched suspension of MS-1 cells.

#### Prussian blue staining of bacterial cells

The PB staining solution for bacterial cells consisted of equal volumes of 2% potassium hexacyanoferrate in distilled water and 5% hydrochloric acid in distilled water (pH = 0.7 in final solution, 0.7 N HCl). A drop of 10 µl magnetically enriched bacteria suspension was pipetted onto an objective slide on a position marked with a diamond engraving pen and allowed to air dry, yielding a film of cells. The film was fixed to the glass surface by immersing the slide in methyl alcohol 99.6% for 15 min, followed by washing three times for 5 min with distilled water. To start dissolving the iron from the magnetosomes, we immersed the slide in 5% hydrochloric acid (1.4 N HCl solution, pH = 0) for 10 min. Slides were incubated in the PB staining solution for 2 h. Variations of the protocol with shorter incubation times did not result in qualitatively different results. After incubation, residues were washed off with distilled water, three times for 5 min each. The samples were dehydrated with a graded alcohol series of 70% ethanol, 96% ethanol, and isopropanol. Glycerol was used to cover the slide-mounted cells for imaging. We produced two series of replicates of MS-1 cells, either subjected to the bacterial PB staining protocol detailed above (10 slides) or to the commercial histological staining kit (Hematognost Fe®, see Methods “Immunohistochemistry and Prussian blue staining and Prussian blue staining of trout olfactory epithelium Sections”) according to the manufacturer’s instructions (10 slides).

For negative controls, we used *Aromatoleum aromaticum* strain EbN1 (Betaproteobacteria, see Rabus et al. 2014^82^), which like *M. magnetotacticum* is Gram-negative, but does not have the biochemical machinery to produce magnetosome chains. To specifically control for potential residues of the ferric quinate as PB-stainable iron, EbN1 cells were immersed for 15 min in the ferric-quinate containing Magnetospirillum growth medium. To control for contamination only, EbN1 cells were not pre-immersed in that medium. Either way, before the application of the PB procedure, EbN1 cells were centrifuged and the pellet was fixed in 4% PFA, followed by washing in distilled water. We prepared two series of replicates (20 slides each) from PB-treated slide-mounted EbN1 cells, with and without prior immersion in ferric quinate containing medium, respectively.

Two samples of magnetotactic bacteria cells were also PB-stained free-floating for detailed investigation under the transmission electron microscope, where we adapted the protocol as follows: All triple washing steps were reduced to one, as a means to limit the loss of cells. After each step the bacteria were centrifuged at 4500 rpm for 20 min.

#### Bright field, confocal reflectance, and enhanced darkfield microscopy

Bright field transmitted light images were taken on a Leica SP2 with a 63 × ApoPlan objective NA 1.32 oil (Leica, Wetzlar, Germany) with an eyepiece CMOS RGB camera (MikroCam SP 5.0, Bresser, Germany). Confocal reflectance scans were acquired with 488 nm illumination, a 70/30 beamsplitter, and the same objective lens.

Enhanced Darkfield images were taken on an upright DMLB microscope (Leica, Wetzlar, Germany) equipped with an Enhanced Darkfield Illuminator (Cytoviva, Auburn, AL, USA), a 63 × ApoPlan objective, NA 1.4-0.6 oil (Leica) and a dark field RGB camera (KA-DF-12S, MikroAge, Germany).

#### Electron microscopy

A 10 µl drop of bacteria solution, either unstained or free-floating stained, was placed on parafilm and a copper grid was placed on top. After 5 min, access liquid was removed by blotting with filter paper. The samples were dried before images were taken on an EM 900 N transmission electron microscope (Zeiss, Oberkochen, Germany). Further electron micrographs, selected area electron diffraction (SAED) patterns, and energy-dispersive X-ray analysis (EDX) maps, were obtained on a JEM2100F TEM at 200 kV (JEOL Ltd., Tokyo, Japan) equipped with two Orius SC200D cameras (Gatan-Ametek, Pleasanton, CA, USA). EDX was performed on an Oxford INCA energy TEM250 EDX system with SDD detector X-Max80 (Oxford Instruments Inc., High Wycombe, UK).

### Ethics approval

Reporting in the manuscript follows the recommendations in the ARRIVE guidelines.

## Supplementary Information

Supplementary Information.

## Data Availability

Data generated and analysed during the current study are included in this submission.
