# Supplementary material for: Prussian blue technique is prone to yield false negative results in magnetoreception research
Source: Sci Rep. 2022 May 25;12:8803. doi: 10.1038/s41598-022-12398-9 (PMC9132912; doi:10.1038/s41598-022-12398-9)
Supplement: Supplementary file 1 — Supplementary Information. [file 41598_2022_12398_MOESM1_ESM.pdf]

Supplementary Material

„Prussian Blue technique is prone to yield false negative results in magnetoreception research“

by Franziska Curdt, Katrin Haase, Laura Ziegenbalg, Helena Greb, Dominik Heyers, Michael Winklhofer.

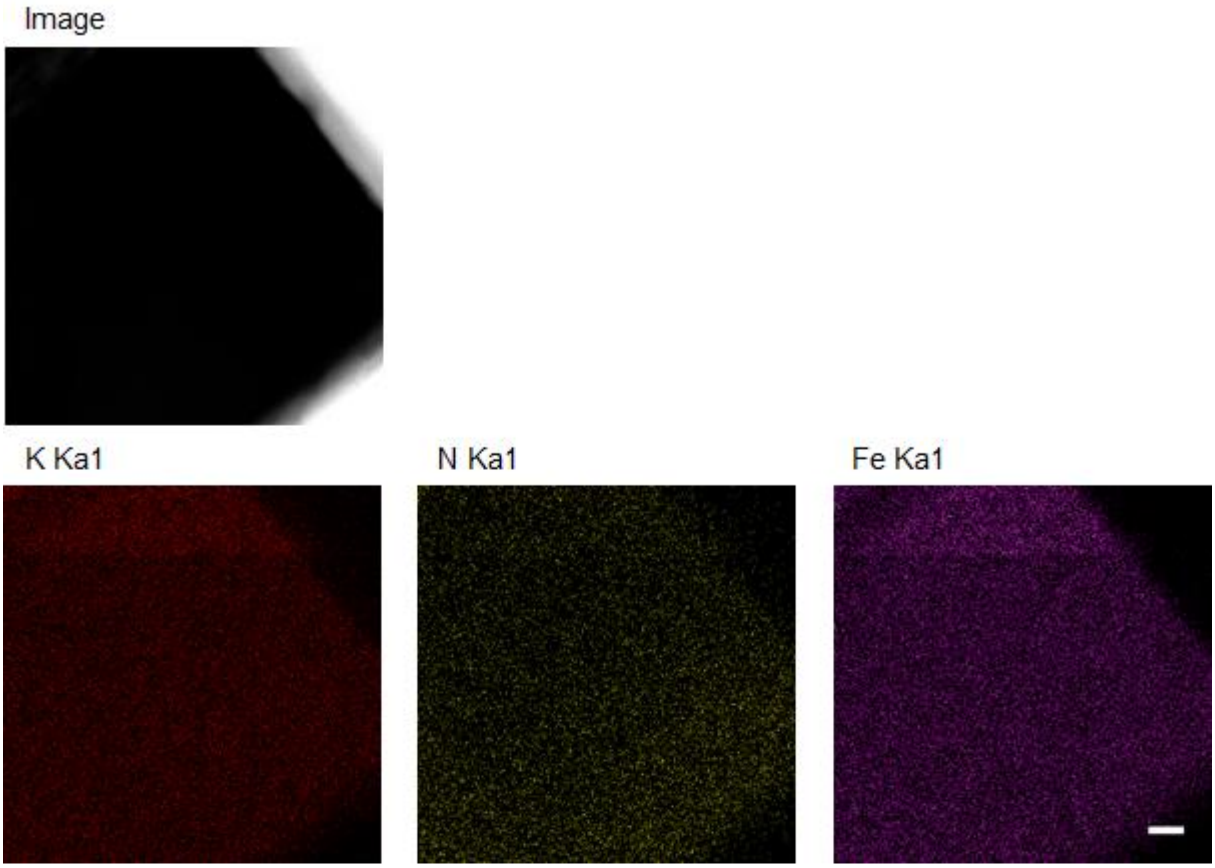

Figure S1 TEM EDX-derived elemental maps of a Prussian blue crystal (see Image top left), showing the spatial distribution of K, N, and Fe, respectively. The co-variation between K and Fe suggests that the Prussian blue crystal does not occur in its pure insoluble form (devoid of K) but rather is a mixed-crystal between soluble and insoluble Prussian blue. Scale bar 30nm.

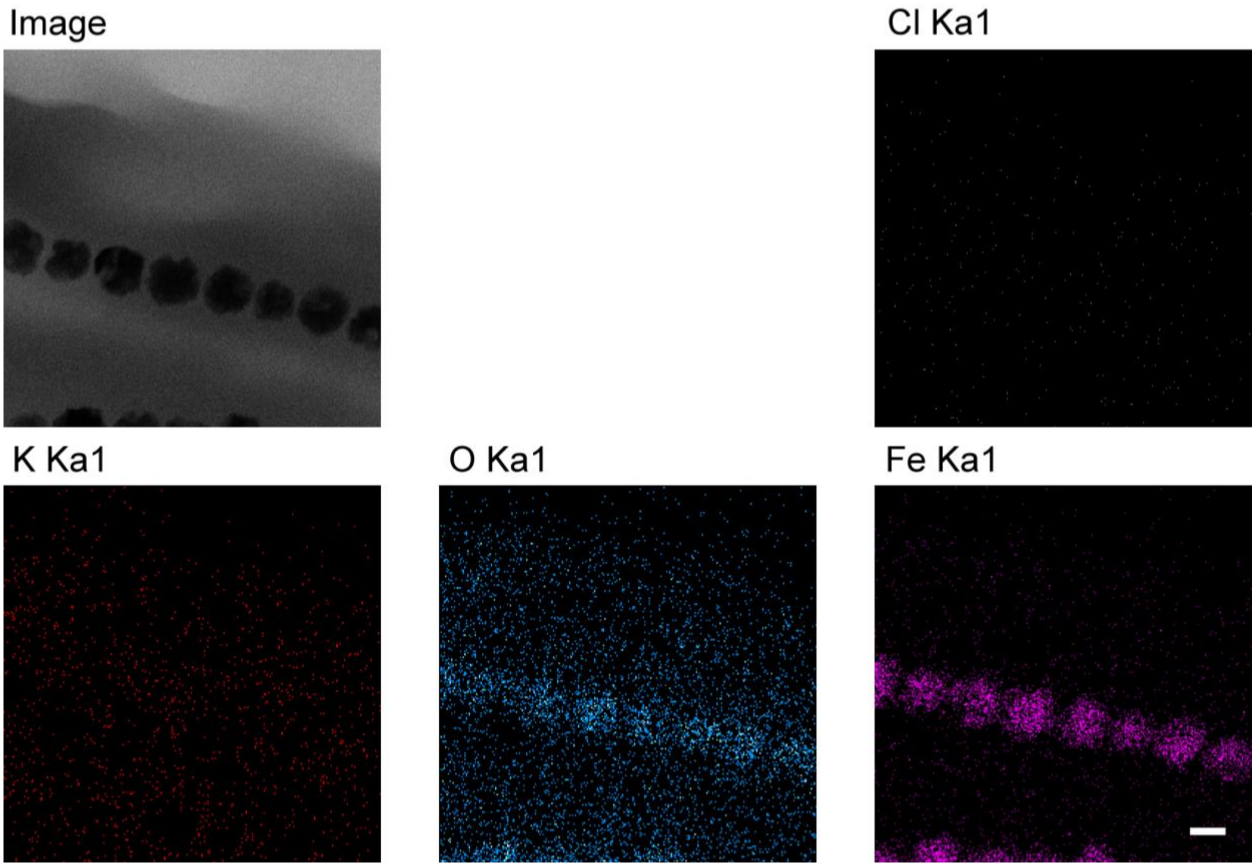

Figure S2 TEM EDX-derived elemental maps of *M. magnetotacticum* after PB staining procedure. Magnetosomes (see image top left) still contain Fe and O, which is incompatible with Prussian Blue. Cl was not detected within cells despite treatment of cells in hydrochloric acid solution as part of PB staining procedure. Scale bar 30nm

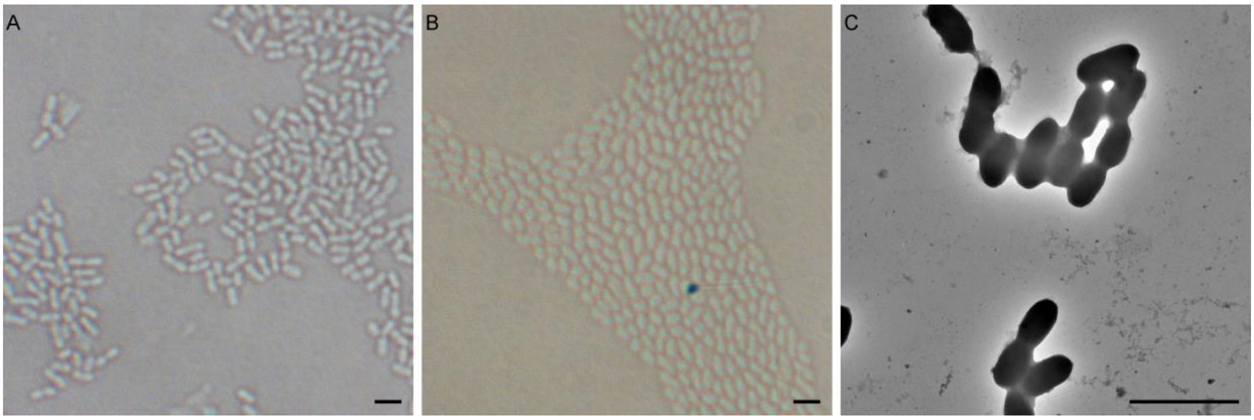

Figure S3 *Aromatoleum aromaticum* EbN1 after Prussian blue staining procedure. Bright field light microscopy (A, B) and TEM (C). In A, cells of EbN1 were pre-immersed in ferric quinate containing Magnetospirillum medium before application of PB procedure. No PB crystals were observed in the entire sample, thus passing the negative-control test. In B, cells of EbN1 were not pre-immersed in ferric quinate containing Magnetospirillum medium before application of PB procedure. Here, a single PB crystal (blue dot in B) was observed on a single slide out of 20 slide-mounted preparations. Scale bars 2 µm.
